# The signaling peptide-encoding genes *CLE16*, *CLE17* and *CLE27* are dispensable for Arabidopsis shoot apical meristem activity

**DOI:** 10.1371/journal.pone.0202595

**Published:** 2018-08-16

**Authors:** Ellen F. Gregory, Thai Q. Dao, Martin A. Alexander, Mark J. Miller, Jennifer C. Fletcher

**Affiliations:** 1 Plant Gene Expression Center, United States Department of Agriculture-Agricultural Research Service, Albany, California, United States of America; 2 Department of Plant and Microbial Biology, University of California Berkeley, Berkeley, California, United States of America; Universidad Miguel Hernández de Elche, SPAIN

## Abstract

The shoot apical meristem produces all of the leaves, stems and flowers of a flowering plant from a reservoir of stem cells at its growing tip. In Arabidopsis, the small polypeptide signaling molecule CLAVATA3 (CLV3), a member of the CLV3/EMBRYO SURROUNDING REGION-RELATED (CLE) gene family, is a key component of a negative feedback loop that maintains stem cell activity in shoot and floral meristems throughout development. Because in some plant species multiple *CLE* genes are involved in regulating shoot apical meristem activity, we tested the hypothesis that *CLE* genes other than *CLV3* might function in stem cell homeostasis in Arabidopsis. We identified three Arabidopsis *CLE* genes expressed in the post-embryonic shoot apical meristem, generated loss-of-function alleles using genome editing, and analyzed the meristem phenotypes of the resulting mutant plants. We found that null mutations in *CLE16*, *CLE17* or *CLE27* affected neither vegetative nor reproductive shoot meristem activity under normal growth conditions, although *CLE27* appears to slightly prolong vegetative growth. Our results indicate that the *CLE16*, *CLE17* and *CLE27* genes have largely redundant roles in the Arabidopsis shoot apical meristem and/or regulate meristem activity only under specific environmental conditions.

## Introduction

Unlike animals, which develop their body plan predominantly during embryogenesis, the distinct architecture of plants is formed throughout the course of their lives. The growing tips of the plant, called the shoot and the root apical meristems, generate organs in a reiterative and continuous process. The shoot apical meristem (SAM) is organized during embryogenesis and produces all of the above ground elements of the plant [1]. Following the germination of the seed, the seedling SAM produces leaves from its flanks during the vegetative phase of development. In response to environmental and endogenous cues the SAM of the mature seedling undergoes the transition to flowering, the reproductive phase in which the shoot meristem is transformed into an inflorescence meristem (IFM) that produces axillary meristems followed by floral meristems that generate the flowers. Fertilization then enables the formation of seeds that transmit the genes to the next generation.

The organization of the SAM provides the capacity for plants to perform lifelong organogenesis. The SAM consists of a small reservoir of stem cells at the apex that is surrounded by a peripheral zone of cells that transition to more differentiated fates within discrete organ primordia. Beneath the stem cell reservoir resides a central domain called the organizing center (OC), which acts as a niche that maintains the fate of the overlying stem cell population. The activity of the OC sustains a relatively constant number of stem cells at the apex of the SAM despite the continuous differentiation of their descendants into organ and stem tissue on the flanks. The spatial and temporal control of gene activity and cellular function within these various domains relies on elaborate networks of phytohormones, transcription factors and intercellular signals to communicate information throughout the shoot apical meristem [2–4].

An intercellular signaling network known as the CLV-WUS pathway maintains stem cell homeostasis in Arabidopsis [5]. The *CLV3* gene is expressed in the stem cells and encodes a small, secreted polypeptide signaling molecule [6] that moves through the apoplast into the cells of the underlying OC, where it is perceived by several receptor kinases complexes [7–11]. Signaling through the CLV pathway restricts the expression of the *WUSCHEL (WUS)* homeobox transcription factor gene to the cells of the OC [12]. WUS protein, in turn, moves through the plasmodesmata into the apical domain [13], where it sustains stem cell identity and directly induces *CLV3* expression [14, 15]. WUS also integrates cytokinin signaling inputs in the interior of the SAM to stimulate cytokinin-mediated stem cell proliferation [16], while repressing the expression of genes that direct cell differentiation [17].

*CLV3* is a founding member of the *CLV3/EMBRYO SURROUNDING REGION-RELATED (CLE)* gene family, which is present throughout the plant lineage and in some plant parasitic nematodes [18, 19]. These genes encode polypeptides of less than 15 kDa in molecular mass that contain an amino-terminal signal peptide, a variable domain, and a conserved stretch of 14 amino acids near the carboxyl-terminus called the CLE domain that is processed to form the biologically active peptide [20–22]. Although the function of the vast majority of *CLE* genes is unknown, studies indicate that CLE peptides play key roles in stem cell homeostasis in Arabidopsis root and vascular meristems as well as in the SAM [23–25]. In some plant species, multiple *CLE* genes appear to be involved in the regulation of stem cell maintenance in shoot and floral meristems [26]. In rice, the *CLV3*-related *FON2* and *FOS1* genes redundantly regulate stem cell activity within floral meristems [27], yet *FON2* also affects inflorescence and axillary meristem maintenance [28] whereas *FOS1* and a third rice *CLE* gene, *FCP1*, are likely to be involved in vegetative SAM maintenance [27, 28]. In tomato, the SlCLV3 and SlCLE9 peptides both appear to affect vegetative meristem size [29], again illustrating potential redundancy within the CLE family. Thus, although they have not been identified in genetic screens, other members of *CLE* gene family may likewise function as additional signaling pathway components in the Arabidopsis SAM.

In this study, we identified three Arabidopsis *CLE* genes that are expressed within the vegetative and/or reproductive SAM. We generated loss-of-function mutations in each of the three genes and analyzed their meristem phenotypes throughout development. We determined that null mutations in the *CLE16*, *CLE17* and *CLE27* genes caused no measurable vegetative, inflorescence or floral meristem phenotypes under normal growth conditions, although CLE27 appears to slightly prolong the vegetative growth rate. Our data suggest that SAM-expressed *CLE* genes other than *CLV3* act largely redundantly in the Arabidopsis meristem and/or function to regulate SAM activity only under certain environmental conditions.

## Materials and methods

### Plant materials and growth conditions

All *Arabidopsis thaliana* plants were in the Columbia-0 accession. The *cle27-2* (SALK_077000) T-DNA insertion allele was generated by the SALK Institute [30] and was obtained from the Arabidopsis Biological Resource Center (ABRC), sequenced to confirm the location of the insertion site, and backcrossed three times to Col-0 prior to analysis. Plants were grown on soil (1:1:1 mixture of perlite:vermiculite:topsoil) under continuous light (120 μmol·m^-2^·s^-1^) at 21°C. Seeds were planted at a density of one seed per pot, except for the Col-0 and *cle16* IFM histology experiment in which a density of two seeds per pot was used. Seeds were stratified at 4°C for 5 days before exposure to light. Seedlings were watered every day with a 1:1500 dilution of Miracle-Gro 20-20-20 fertilizer prior to flowering and once a week with fertilizer thereafter. Homozygous mutant plants were confirmed by PCR-based genotyping prior to analysis (primers listed in S1 Table).

### Genome editing of *CLE* gene loci

CRISPR-Cas9 target gene sequences for *CLE16* and *CLE17* were identified using the CRISPR-P website [31]. The target sequences were amplified and cloned into the sgRNA cassette of the Gateway-compatible pSGR_pGEMT entry vector, which also harbored a Cas9 expression cassette. The pSGR_pGEMT constructs containing the Cas9 cassette as well as the *CLE16* or *CLE17* genomic target sequences were transferred into the pEarleyGate 301 binary vector using the LR enzyme mix (ThermoFisher Scientific), and sequenced. The recombinant pEarleyGate 301 constructs were then transferred into *Agrobacterium tumefaciens* GV3101 and transformed into wild-type Col-0 plants using the floral dip method [32]. The T1 seeds were sown and selected by spraying twice with 0.01% BASTA solution, 3–5 days apart. Resistant transformants were genotyped using the Cleaved Amplified Polymorphic Sequence (CAPS) method with gene-specific primers (primers listed in S1 Table). Heterozygous T1 mutant plants were self-fertilized and homozygous T2 individuals identified by genotyping, followed by sequencing to confirm the mutant allele.

Genotyping the *CLE16* CRISPR alleles was performed by using forward and reverse primers (primers listed in S1 Table) in a Polymerase Chain Reaction (PCR) to amplify a 995 bp product. Digesting the PCR product with MspI yielded 779 bp and 216 bp bands from wild-type tissue, whereas the product from mutant tissue remained undigested. Genotyping the *CLE17* CRISPR alleles was performed by using forward and reverse primers in a PCR reaction to amplify a 1016 bp product. Digesting the PCR product with BslI yielded 770 bp, 233 bp and 13 bp bands from wild-type tissue, whereas the product from mutant tissue remained undigested. Genotyping to confirm the absence of the Cas9 cassette from *cle16* and *cle17* mutant plants was performed using Cas9 forward and reverse primers (primers listed in S1 Table).

### Phenotypic analysis

Whole seedlings, rosette leaves, inflorescences and flower specimens were imaged using Zeiss Stemi 2000-c and Zeiss Stemi SV11 microscopes, and images were acquired using a Canon D-40 digital camera. Inflorescence apices were prepared for scanning electron microscopy as described [33] and visualized on a Hitachi S4700 scanning electron microscope. Inflorescence apices were prepared for histology as described [34], stained for 25 seconds in a 0.1% Toluidine blue 0 dye solution (Sigma), de-stained through an ethanol series, and sectioned at 4 μm thickness. Sections were visualized using a Zeiss Axiovert 200M microscope. Floral organ counting was performed as described [33].

## Results

The starting point for our functional analysis was the identification of all *CLE* genes expressed in the SAM during vegetative or reproductive development. For the vegetative stage, we used promoter:GUS expression data gathered from the vegetative meristems of 10-day-old seedlings [35]. These data indicated that, in addition to *CLV3*, the promoters of both *CLE16* and *CLE17* drove expression in the vegetative meristem as well as in the adjacent organ primordia, although the pCLE16:*GUS* signal was much weaker than the pCLE17:*GUS* signal in the SAM itself [35]. For the reproductive stage, we mined published transcriptome data generated from laser micro-dissected IFMs [36] for *CLE* gene expression. The *CLV3* gene was used as a positive control and appeared with an expression value of 31.27 RPKM (S1 Fig). In addition, the *CLE17*, *CLE20*, *CLE27* and *CLE42* genes were all detected as being expressed in the IFMs transcriptome dataset (S1 Fig). Among these, we omitted *CLE20* from our analysis because our promoter:GUS data indicated that the promoter drove expression exclusively in the vasculature, including in the vascular strands directly beneath the SAM, but not within the SAM itself [35]. We also excluded *CLE42* because a previous study reported that a loss-of-function *cle42* T-DNA insertion allele displayed no shoot phenotype [37]. Consequently we focused on the functional analysis of the *CLE16*, *CLE17* and *CLE27* genes during Arabidopsis shoot development.

### Generation of *cle* loss-of-function alleles

Two independent loss-of-function alleles of each of the three *CLE* genes were identified for functional characterization. Although *CLE* gene loci represent small targets for mutagenesis, a single allele of both *CLE16* and *CLE17* had already been reported (Table 1). The *cle16-1* Ds transposon insertion in the *CLE16* coding region acts as a transcriptional null allele; however, the genetic background is the Nossen accession [35]. The T-DNA insertion in the *cle17-1* Col-0 allele is located in the 3’ untranslated region (UTR) downstream of the *CLE17* coding region, and behaves as a hypomorphic, partial loss-of-function allele rather than a null allele [35].

**Table 1:** Alleles of *CLE* genes expressed in the shoot apical meristem.

CLE Gene	Mutant Allele	Type of Mutation	Source
*CLE16*	*cle16-1*	Ds transposon insertion at +37 bp	Jun et al 2010
*CLE16*	*cle16-2*	Insertion of “C” nucleotide at +59 bp	This work
*CLE16*	*cle16-3*	Deletion of “G” nucleotide at +59 bp	This work
*CLE17*	*cle17-1*	T-DNA insertion at +412 bp	Jun et al 2010
*CLE17*	*cle17-2*	Insertion of “A” nucleotide at +220 bp	This work
*CLE17*	*cle17-3*	Insertion of “T” nucleotide at +220 bp	This work
*CLE27*	*cle27-1*	T-DNA insertion at +149 bp	This work
*CLE27*	*cle27-cr1*	Insertion of “T” nucleotide at +173 bp	Yamaguchi et al 2017

**Fig 1:**
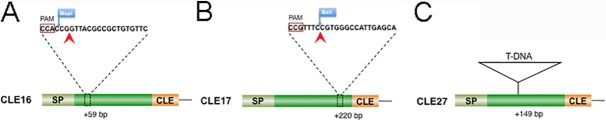
Graphic representation of mutations in the *CLE16*, *CLE17* and *CLE27* genes. (A) Location of the *cle16* CRISPR-Cas9 induced mutations (red arrowhead) relative to the sgRNA PAM site and MspI restriction site in the *CLE16* coding sequence. (B) Location of the *cle17* CRISPR-Cas9 induced mutations (red arrowhead) relative to the sgRNA PAM site and BslI restriction site in the *CLE17* coding sequence. (C) Location of the *cle27-2* T-DNA insertion in the *CLE27* coding sequence. SP, signal peptide sequence; CLE, CLE domain sequence.

Because *CLE16* and *CLE17* null alleles in the Col-0 accession were unavailable for comparative analysis, we generated new loss-of-function alleles of the two genes using CRISPR-Cas9 genome engineering (Table 1 and Fig 1). Transformation of wild-type Col-0 plants with an sgRNA targeted to the *CLE16* coding sequence yielded multiple independent transformants. We detected 21 mutant individuals among the 24 T1 plants analyzed, a remarkable 87.5% mutation rate. Mutations in the T1 individuals were made homozygous in the T2 generation and confirmed by sequencing, and two were chosen for further study. One line contained an insertion of a “C” nucleotide after position +59 downstream of the translation start site (Fig 1A), and was designated *cle16-2*. A second line contained a deletion of a “G” nucleotide after position +59 and was designated *cle16-3*. Each of these mutations generates a frame shift in the *CLE16* coding sequence well upstream of the CLE domain, with the *cle16-2* mutation also introducing several premature stop codons.

Transformation with an sgRNA targeted to the *CLE17* coding sequence also yielded multiple independent transformants. We detected 5 mutant individuals among the 24 T1 plants analyzed, a 20.1% mutation rate. Mutations in the T1 individuals were made homozygous in the T2 generation and confirmed by sequencing, and two were chosen for further study. One line contained an insertion of an “A” nucleotide after position +220 downstream of the translation start site (Fig 1B), and was designated *cle17-2*. A second line contained an insertion of a “T” nucleotide after position +220, and was designated *cle17-3*. Each of these mutations generates a frame shift that introduces a premature stop codon in the *CLE17* coding sequence upstream of the CLE domain. Due to the frame shift mutations none of these *cle16* or *cle17* alleles produces a functional CLE polypeptide, and thus they represent loss-of-function alleles.

In addition we identified a *CLE27* T-DNA insertion allele in the Col-0 accession from the SALK collection [30], which to avoid confusion with the published CRISPR/Cas9 line described below we designate *cle27-2*. Sequencing indicated that *cle27-2* carries a T-DNA insertion +149 base pairs (bp) downstream of the translation start site (Fig 1C), in the center of the *CLE27* coding region (Table 1). The insertion site is located upstream of the *CLE27* CLE domain, indicating that *cle27-2* represents a loss-of-function allele. A second, independent *CLE27* allele used was a CRISPR/Cas9-generated loss-of-function allele in the Col-0 accession designated *cle27-cr1* [38]. This allele generates a frame shift that introduces a premature stop codon in the *CLE27* coding sequence upstream of the CLE domain (Table 1), indicating that it is a null allele [38].

### Analysis of SAM function during vegetative development

**Fig 2:**
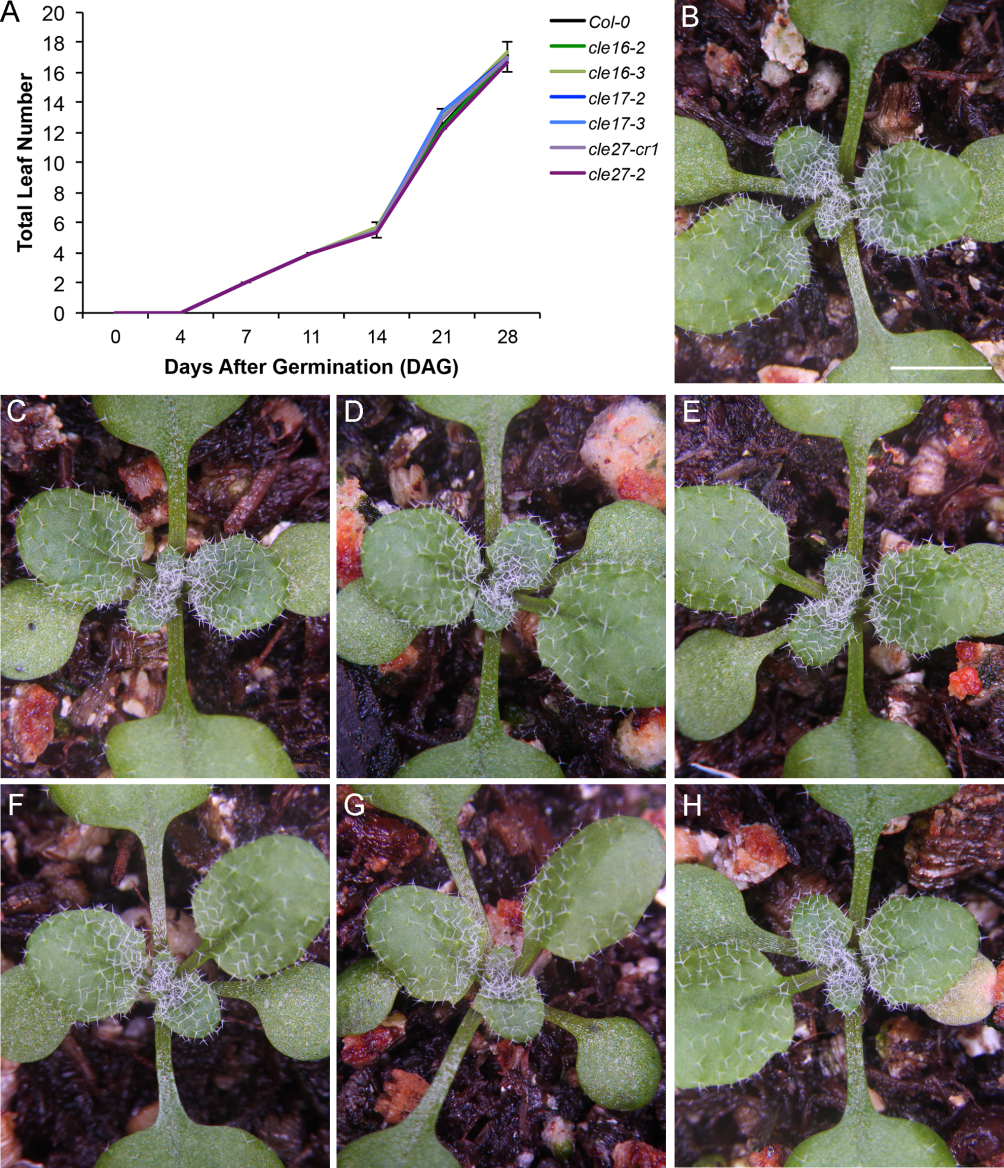
Mutations in *CLE16*, *CLE17* or *CLE27* have no effect on the leaf initiation rate of the shoot apical meristem. (A) Leaf initiation rate in wild-type and *cle* mutant plants from 1 to 28 days after germination (DAG). Values shown are mean ± standard deviation (S.D.). n = 12 individuals per genotype. (B) Wild-type Col-0 rosette at 14 DAG. (C) *cle16-2* rosette. (D) *cle16-3* rosette. (E) *cle17-2* rosette. (F) *cle17-3* rosette. (G) *cle27-cr1* rosette. (H) *cle27-2* rosette. Scale bar, 1 cm.

To determine whether the *CLE16*, *CLE17* or *CLE27* genes play a role in regulating shoot apical meristem activity during vegetative development, we analyzed the phenotypes of wild-type Col-0 as well as *cle16-2*, *cle16-3*, *cle17-2*, *cle17-3*, *cle27-cr1* and *cle27-2* seedlings from germination through the first four weeks of growth. We first measured the rate of rosette leaf initiation from the SAM beginning one day after germination (DAG), then again at 4 and 7 DAG, and weekly thereafter (Fig 2A). We found that all of the wild type and *cle* mutant seedlings had produced two rosette leaves between 4 and 7 DAG, and that by 14 DAG plants of all genotypes had produced an average of 5–6 rosette leaves (Fig 2A). The arrangement and morphology of the leaves was indistinguishable between the various genotypes at this stage of seedling development (Fig 2B–2H). The rate of leaf initiation from the SAM was not significantly different between Col and *cle16*, *cle17* or *cle27* seedlings during the first 28 days of vegetative growth (Fig 2A), after which time the plants began to undergo the transition to flowering. These results indicate that neither *CLE16*, *CLE17* nor *CLE27* individually functions in regulating SAM activity during the early stages of Arabidopsis vegetative development.

**Fig 3:**
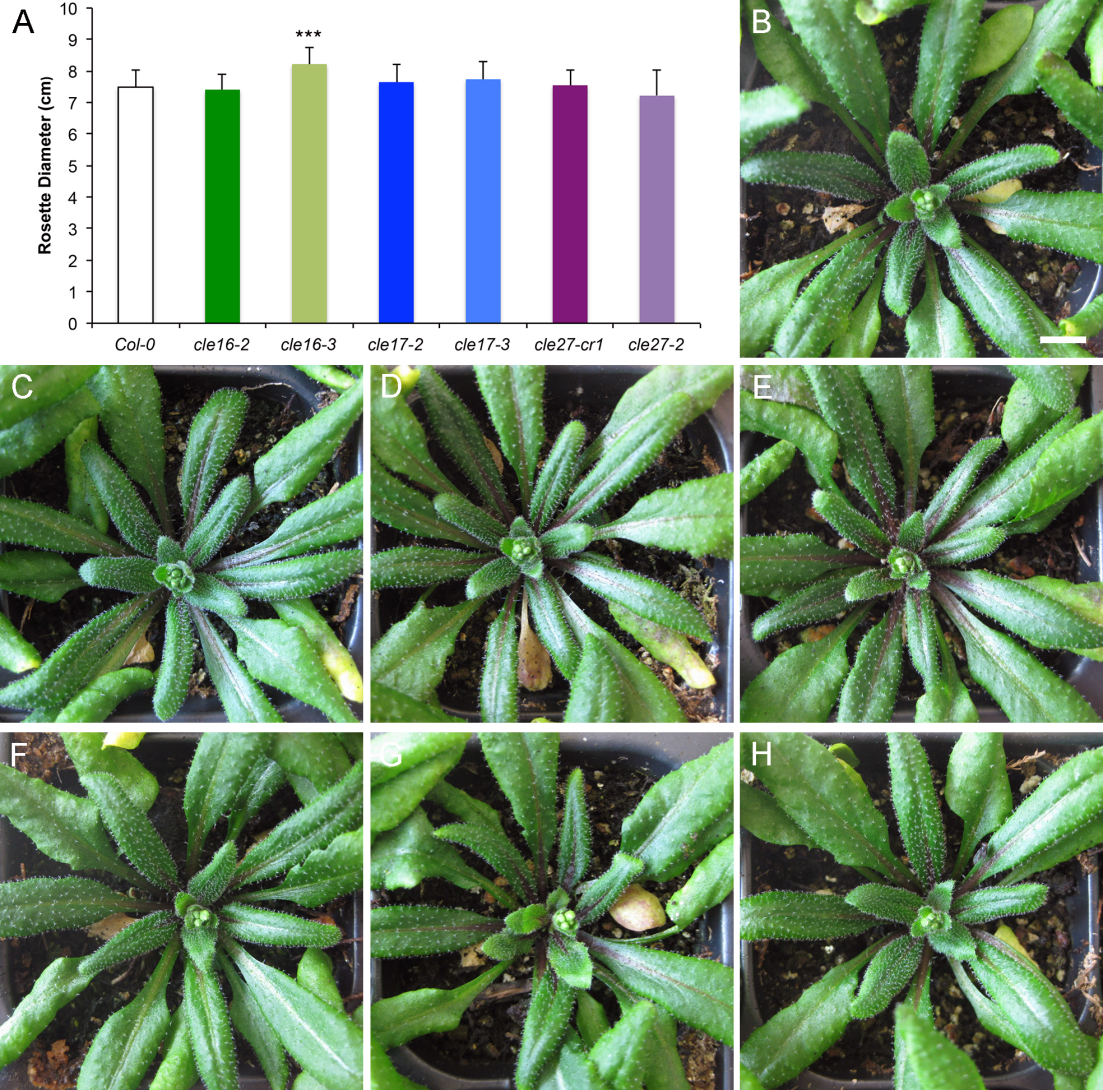
Mutations in *CLE16*, *CLE17* or *CLE27* have no effect on the rosette diameter of mature seedlings. (A) Rosette diameter of wild-type and *cle* mutant plants at the floral transition. Values shown are mean ± standard deviation (S.D.). Asterisks indicate a significant difference from wild-type at p <0.001 (two-tailed Student’s t test). n = 14–20 individuals per genotype. (B) Wild-type Col-0 rosette at the floral transition. (C) *cle16-2* rosette. (D) *cle16-3* rosette. (E) *cle17-2* rosette. (F) *cle17-3* rosette. (G) *cle27-cr1* rosette. (H) *cle27-2* rosette. Scale bar, 1 cm.

Next we quantified the rosette diameter of wild-type and *cle* mutant plants at the floral transition, when the mature seedlings ceased producing vegetative organs. Compared with wild-type Col-0 plants (Fig 3A and 3B), the diameter of *cle17* rosettes (Fig 3A, 3E and 3F) and *cle27* rosettes (Fig 3A, 3G and 3H) was unaltered. The diameter of *cle16-3* rosettes was slightly but significantly larger than those of Col-0 and *cle16-2* rosettes (Fig 3A, 3C and 3D); however, that of *cle16-2* plants was indistinguishable from the wild type (Fig 3A and 3C). Because only one of the two *cle16* null alleles has this effect we conclude that neither *CLE16*, *CLE17* nor *CLE27* is likely to play an independent role in rosette growth.

A major developmental event that alters the activity of the shoot apical meristem is the floral transition. This is when the SAM integrates endogenous signals as well as environmental signals from the leaves into broad transcriptional alterations that change the identity of the meristem from vegetative to reproductive. The reproductive, or inflorescence meristem, then initiates a number of axillary meristems followed by floral meristem primordia from its flanks. To determine whether the SAM-expressed *CLE* genes played any role in the transition of the meristem from vegetative to reproductive activity, we measured the number of days to bolting, total leaf number and axillary meristem number in wild-type and *cle* mutant plants.

**Fig 4:**
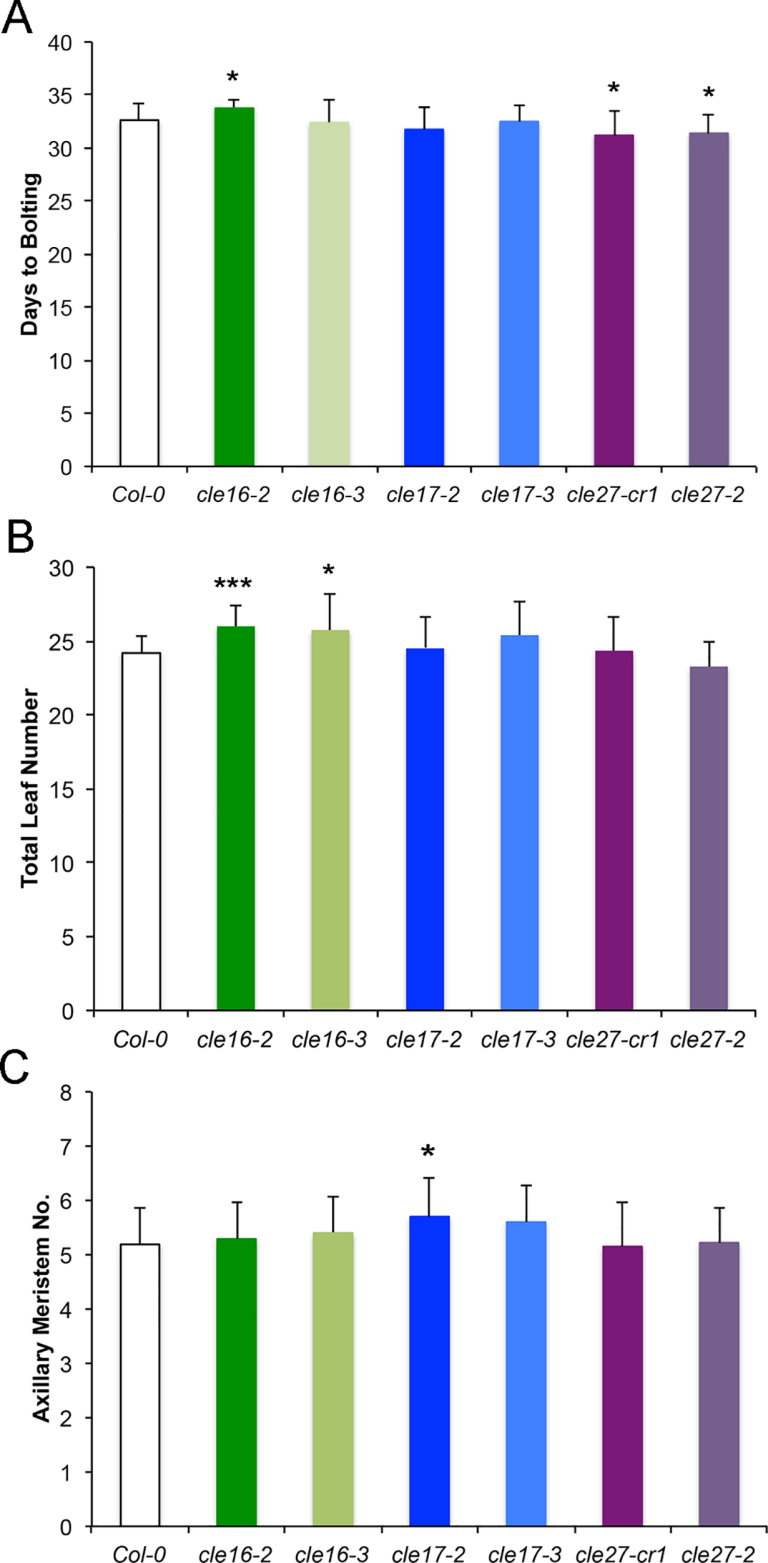
Mutations in *CLE16*, *CLE17* or *CLE27* have no significant effect on the floral transition. (A) Days to bolting of wild-type and *cle* mutant plants. (B) Total leaf number of wild-type and *cle* mutant plants at the transition to flowering. (C) Axillary meristem number in wild-type and *cle* mutant plants. Values shown in each graph are mean ± standard deviation (S.D.). Asterisks indicate a significant difference from wild-type at * p < 0.05; *** p <0.001 (two-tailed Student’s t test). n = 16–20 individuals per genotype.

We observed no difference in either mean days to bolting or total leaf number in *cle17* plants compared to wild-type Col-0 plants (Fig 4A and 4B), indicating that *CLE17* activity does not affect the floral transition. We detected a small decrease in the number of days to bolting in plants homozygous for either *cle27* allele. Both *cle27-cr1* and *cle27-2* plants flowered an average of one day earlier than wild-type when grown under constant light conditions: 32.63±1.63 days for Col-0 compared to 31.2±2.29 for *cle27cr-1* and 31.39±1.79 days for *cle27-2* plants (Fig 4A). However, the total number of leaves at flowering was unchanged in *cle27-cr1* and *cle27-2* plants (Fig 4B), suggesting that *CLE27* may slightly prolong the plant growth rate over time rather than specifically affecting the floral transition [39]. Conversely, *cle16-2* and *cle16-3* plants both generated one to two more leaves than wild-type plants prior to flowering, and the *cle16-2* allele also slightly delayed the time to bolting (Fig 4A and 4B). However, an independent experiment performed using identical growth conditions showed no significant difference between the two *cle16* alleles and wild-type Col-0 with respect to either days to bolting or total leaf number (S2 Fig). Thus these data indicate that *CLE16*, *CLE17* and *CLE27* have no significant effect on the transition to flowering under constant light conditions.

During the transition to flowering the Arabidopsis primary SAM produces a small number of axillary meristems from the axils of the cauline leaves. Under our growth conditions wild-type Col-0 plants generated an average of 5.2±0.66 axillary meristems per SAM (Fig 4C). Neither *cle16* nor *cle27* plants displayed altered axillary meristem number (Fig 4C). A very slight increase in axillary meristem number, to an average of 5.7±0.73, was detected in *cle17-2* plants (Fig 4C). However, because such an increase was not observed in *cle17-3* plants we conclude that *CLE17* also has no significant effect on axillary meristem formation. These results indicate that neither *CLE16*, *CLE17* nor *CLE27* contributes to regulating the process of axillary meristem formation by the shoot apical meristem.

### Analysis of SAM function during reproductive development

**Fig 5:**
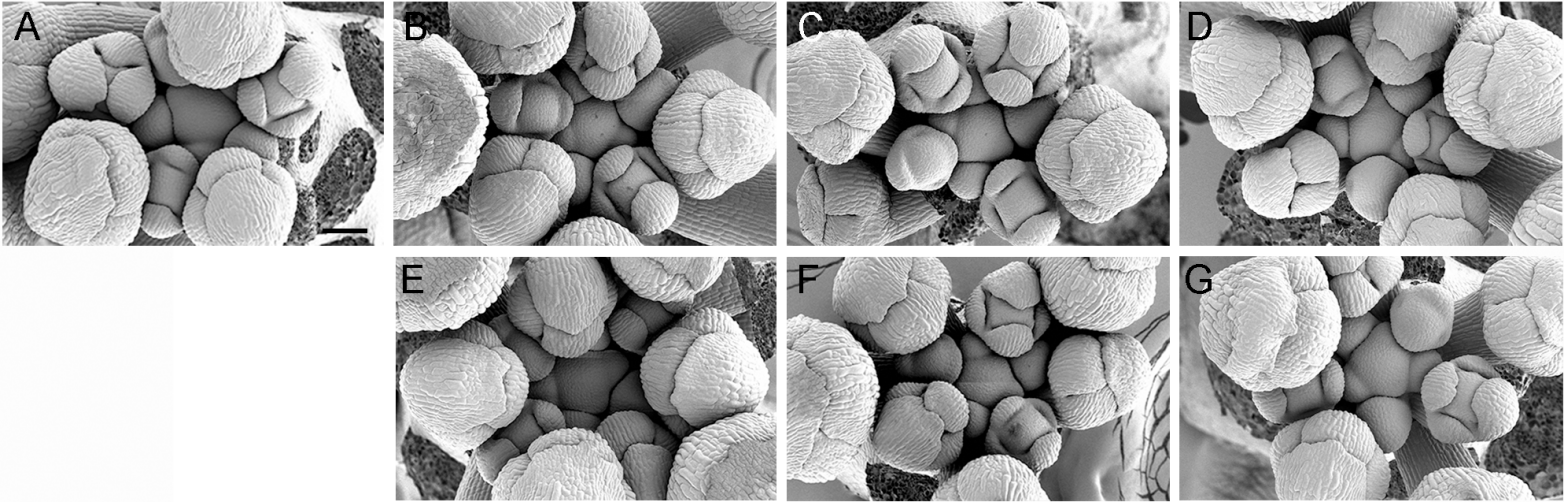
Mutations in *CLE16*, *CLE17* or *CLE27* have no effect on inflorescence meristem morphology or phyllotaxy. (A) Col-0 IFM. (B) *cle16-2* IFM. (C) *cle16-3* IFM. (D) *cle17-2* IFM. (E) *cle17-3* IFM. (F) *cle27-cr1* IFM. (G) *cle27-2* IFM. Scale bar, 50 μm.

Next we determined whether the *CLE16*, *CLE17* or *CLE27* genes functioned in regulating shoot apical meristem activity during reproductive development by comparing inflorescence and floral meristem activity between wild-type Col-0 and *cle16*, *cle17*, and *cle27* plants. It is known that *clv3* plants form enlarged inflorescence meristems that produce many more flowers than wild-type plants in a random rather than a spiral phyllotaxy [40]. Using scanning electron microscopy, we examined the tips of wild-type and *cle* mutant inflorescence meristems harvested when the length of the stem reached 1 cm. The morphology of the *cle16*, *cle17* and *cle27* IFMs was indistinguishable from that of wild-type Col-0 inflorescences, as was the rate of floral meristem initiation (Fig 5). The phyllotaxy, or arrangement, of floral meristem formation from the IFM flanks was also unaffected, with successive floral primordia initiating in a spiral pattern in both wild-type and *cle* mutant plants (Fig 5).

**Fig 6:**
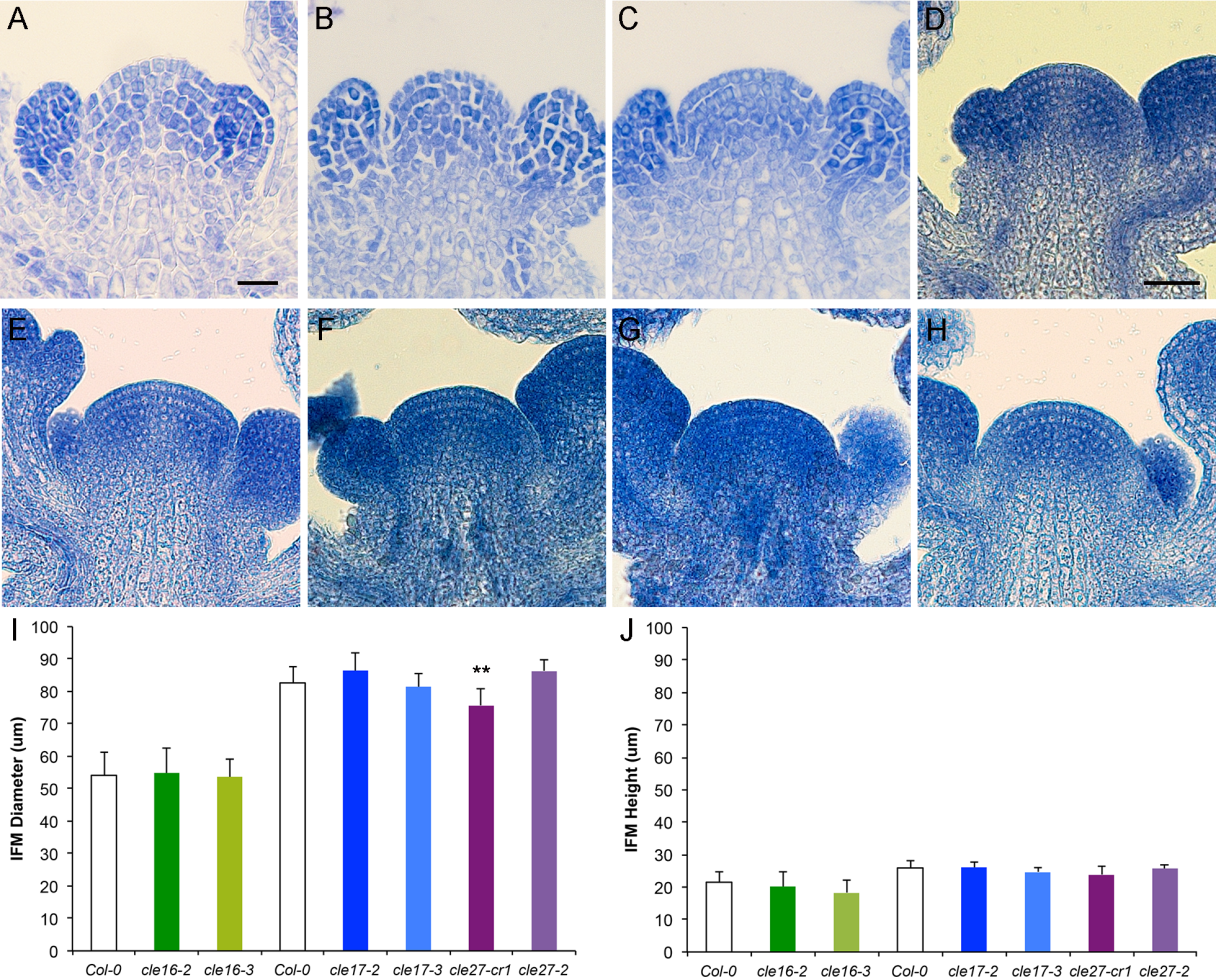
Mutations in *CLE16*, *CLE17* or *CLE27* have no effect on inflorescence meristem size. (A-H) Longitudinal section through an (A) Col-0 IFM, (B) *cle16-2* IFM, (C) *cle16-3* IFM, (D) Col-0 IFM, (E) *cle17-2* IFM, (F) *cle17-3* IFM, (G) *cle27-cr1* IFM, (H) *cle27-2* IFM. (I, J) Inflorescence meristem diameter (I) and height (J) in wild-type and *cle* mutant plants. Values shown in each graph are mean ± standard deviation (S.D.). n = 10–17 individuals per genotype. Asterisks indicate a significant difference from wild-type at ** p < 0.01 (two-tailed Student’s t test). Scale bar, 20 μm for A-C and 50 μm for D-H.

We analyzed wild-type and *cle* inflorescence meristem morphology and size in greater detail by histological sectioning. The wild-type Col-0 inflorescence meristem is dome-shaped and is composed of three cell layers (Fig 6A and 6D). The cells in the outermost two cell layers, L1 and L2, divide in a strictly anticlinal orientation and form the epidermal and sub-epidermal layers [1], respectively. The underlying L3 cells divide in all orientations and provide the girth of the IFM. The morphology of *cle16*, *cle17* and *cle27* IFMs was indistinguishable from that of wild-type IFMs, and the layering of the meristem was intact in all genotypes (Fig 6B, 6C and 6E–6H). *clv3* mutant inflorescence meristems contain many more cells and are both wider and taller than wild-type IFMs [12], so the diameter and height of Col-0, *cle16*, *cle17* and *cle27* IFMs was measured. Two experiments were performed, one comparing *cle16* homozygous IFMs to Col-0 and the other comparing *cle17* and *cle27* IFMs to Col-0. Although the mean Col-0 IFM size differed between the two experiments due to slightly different cultivation conditions (see Methods), the mean size of the *cle16*, *cle17* and *cle27* IFMs was not significantly different from that of the corresponding Col-0 IFMs (Fig 6I and 6J). These observations show that, unlike *CLV3*, *CLE16*, *CLE17* and *CLE27* individually have no effect on inflorescence meristem activity under normal growth conditions.

**Fig 7:**
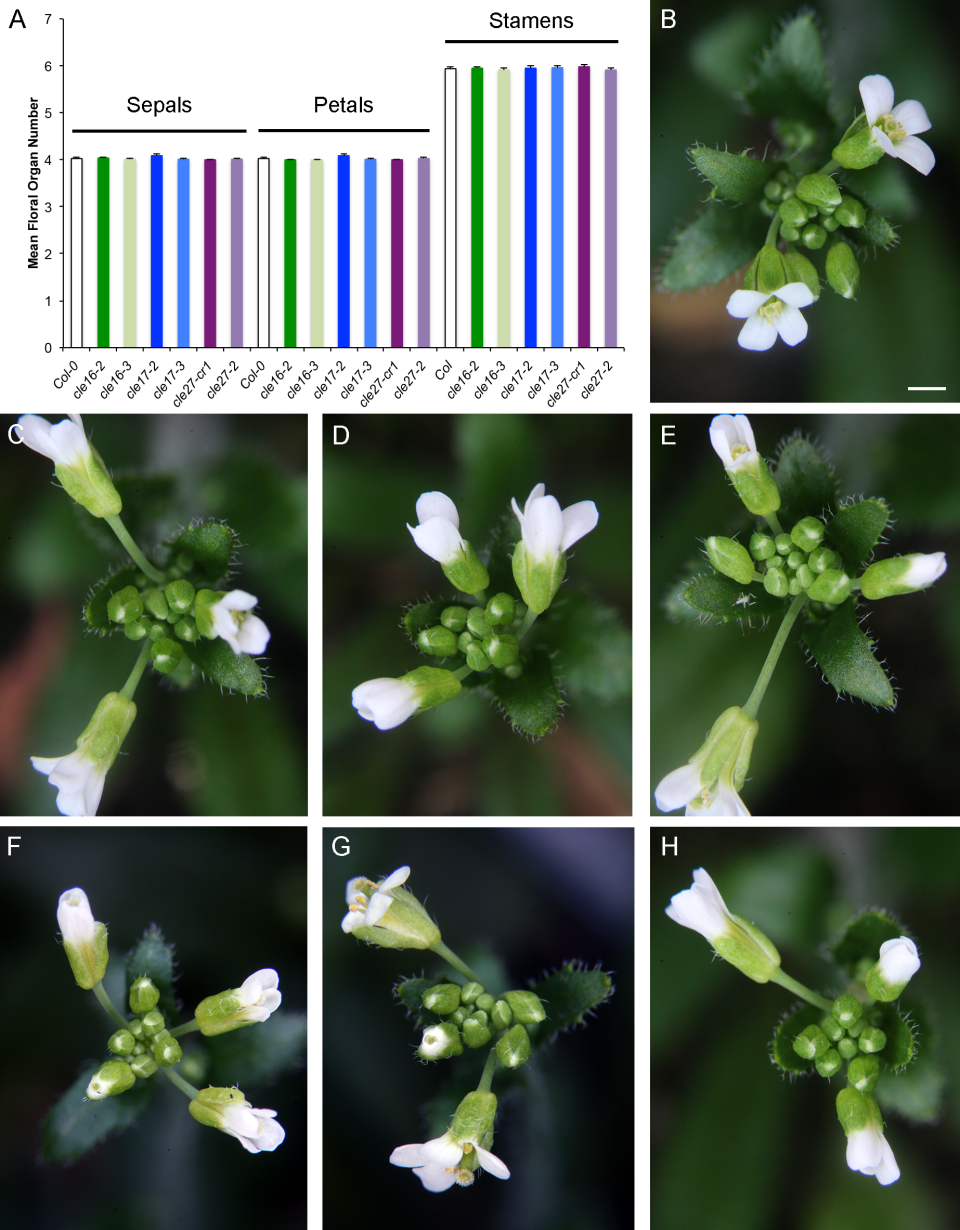
Mutations in *CLE16*, *CLE17* or *CLE27* do not affect floral organ number. (A) Floral organ number in wild-type and *cle* mutant plants. Values shown are mean ± standard error (S.E.). n = 80 flowers per genotype. (B) Col-0 buds and open flowers. (C) *cle16-2* buds and open flowers. (D) *cle16-3* buds and open flowers. (E) *cle17-2* buds and open flowers. (F) *cle17-3* buds and open flowers. (G) *cle27-cr1* buds and open flowers. (H) *cle27-2* buds and open flowers. Scale bar, 0.5 cm.

Finally, we quantified the number of floral organs in wild-type and *cle* mutant flowers as a readout for potential alterations in floral meristem size. Compared to wild-type flowers, which consist of four sepals in the first whorl, four petals in the second whorl, 5–6 stamens in the third whorl and two carpels in the fourth whorl (Fig 7A and 7B), *clv3* flowers produce supernumerary organs in all four whorls, particularly the inner two, and can generate additional organs within the carpel whorl [6, 40]. The extra floral organs are a product of enlarged floral meristems and the extra whorls of organs result from reduced floral meristem determinacy [40]. In contrast to *clv3* mutants, the mean number of sepals, petals and stamens produced by plants carrying null mutations in *CLE16*, *CLE17* or *CLE27* was indistinguishable from the wild type (Fig 7A and 7C–7H). Carpel number in all genotypes was invariant at two. These data indicate that individually *CLE16*, *CLE17* and *CLE27* are dispensable for regulating floral meristem activity.

## Discussion

The aim of our study was to identify additional members of the Arabidopsis *CLV3*-related *CLE* gene family that are expressed in above-ground meristems and to determine whether they played a role in regulating shoot apical meristem activity. We identified *CLE16* and *CLE17* as being expressed in both vegetative and inflorescence meristems, and *CLE27* as being expressed in inflorescence meristems and vegetative leaf primordia. Our functional analysis indicates that loss-of-function mutations in *CLE16*, *CLE17* or *CLE27* have no significant effect on vegetative leaf initiation rate or rosette diameter, on inflorescence meristem morphology, phyllotaxy or size, or on floral organ number. *CLE27* does, however, seem to play a small role in prolonging the vegetative growth rate, as *cle27* plants flower on average one day earlier than wild-type plants (Fig 4). Thus unlike *CLV3*, the *CLE16*, *CLE17* and *CLE27* genes are largely dispensable for shoot apical meristem maintenance on their own. Consistent with this result, none of the three genes activates the *CLV3* signaling pathway when over-expressed in the SAM [35].

Comparison of the three CLE peptides with CLV3 reveals differences at key residues. The CLE16 and CLE17 peptides are identical except at position 2 [41], and both differ from the CLV3 peptide at several residues, including the C-terminal histidine residue that has an essential role in CLV3 peptide function and binding to the receptor kinase CLV1 [42]. The CLE27 peptide is also divergent, differing from CLV3 at the 2^nd^ and 12^th^ positions and also containing a cysteine residue in place of the highly conserved glycine residue at position 6 that when mutated in CLV3 causes a moderate stem cell accumulation phenotype [6]. These observations suggest that the CLE16, CLE17 and CLE27 peptides are not perceived by the receptor kinase complexes that interact with CLV3, and instead may have functions within the SAM that are not related to maintaining stem cell homeostasis via the CLV-WUS pathway.

There may be several reasons why, unlike *CLV3*, the *CLE16*, *CLE17* and *CLE27* genes individually have no discernable developmental phenotypes under standard growth conditions. One possibility is that the three *CLE* genes have redundant functions in Arabidopsis meristems. The *CLE* gene family consists of 32 members in Arabidopsis [18, 43], only a few of which exhibit single mutant phenotypes [24, 35, 37, 44]. In addition, most Arabidopsis tissues express multiple *CLE* genes in overlapping patterns [35], and many CLE peptides act interchangeably when ectopically expressed in roots or shoots [20, 43, 45, 46]. These observations suggest that *CLE* gene functional redundancy may be widespread. Consistent with this notion, we have observed no meristem-related phenotypes among *cle16 cle17*, *cle16 cle27*, or *cle17 cle27* double mutant plants. Therefore generating even higher order mutant combinations among SAM-expressed *CLE* genes may be required to uncover meristem-related phenotypes. An important corollary to our study is that targeting the orthologous *CLE16*, *CLE17* or *CLE27* genes one by one in agricultural plant species is unlikely to be sufficient to enhance yield, but that targeting the genes in combination may prove a more effective strategy for crop improvement to benefit agricultural productivity.

A second, and non-exclusive, explanation for the absence of phenotypes is that the three *CLE* genes regulate SAM activity only under specific environmental conditions. To date only a handful of studies describing the effect of different environmental states on Arabidopsis *CLE* gene activity have been published. In roots, induction of *CLE14* expression under phosphorus limiting conditions causes terminal differentiation of the root apical meristem [47], while a CLE-CLV1 signaling module is proposed to prevent the expansion of the lateral root system in nitrogen-poor environments [48]. Above ground, *CLE45* has been shown to play a role in prolonging pollen tube growth only at high temperatures [49]. With the recent availability of null alleles for all Arabidopsis *CLE* genes generated using genome-editing technology [38], we are rapidly developing the tools needed to determine the significance of these small but important signaling molecules for Arabidopsis biology.

## Supporting information

S1 Fig Mean *CLE* gene expression levels in inflorescence meristems (IM). (TIF)

S2 Fig Mutations in *CLE16* have no significant effect on the floral transition. (TIF)

S1 Table Primer sequences used in the study. (DOCX)
