# Supplementary figures and images for: The signaling peptide-encoding genes CLE16, CLE17 and CLE27 are dispensable for Arabidopsis shoot apical meristem activity
Source: PLoS One. 2018 Aug 16;13(8):e0202595. doi: 10.1371/journal.pone.0202595 (PMC6095548; doi:10.1371/journal.pone.0202595)

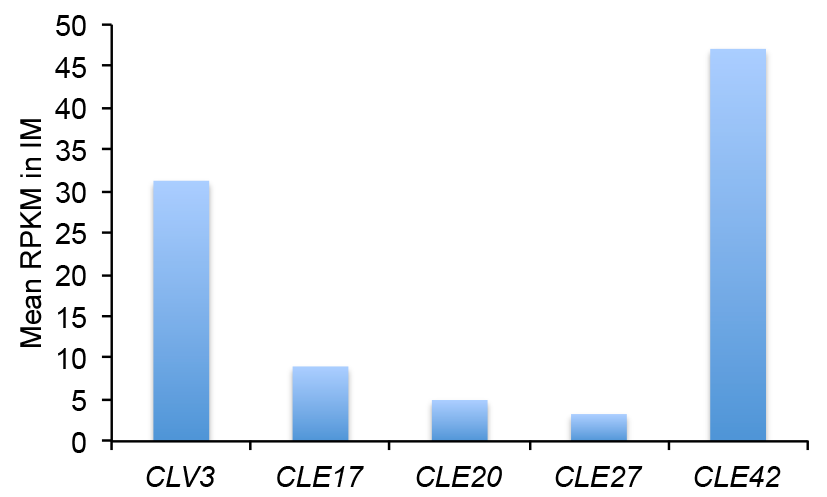

Supplement: S1 Fig — (TIF) [file pone.0202595.s001.tif]

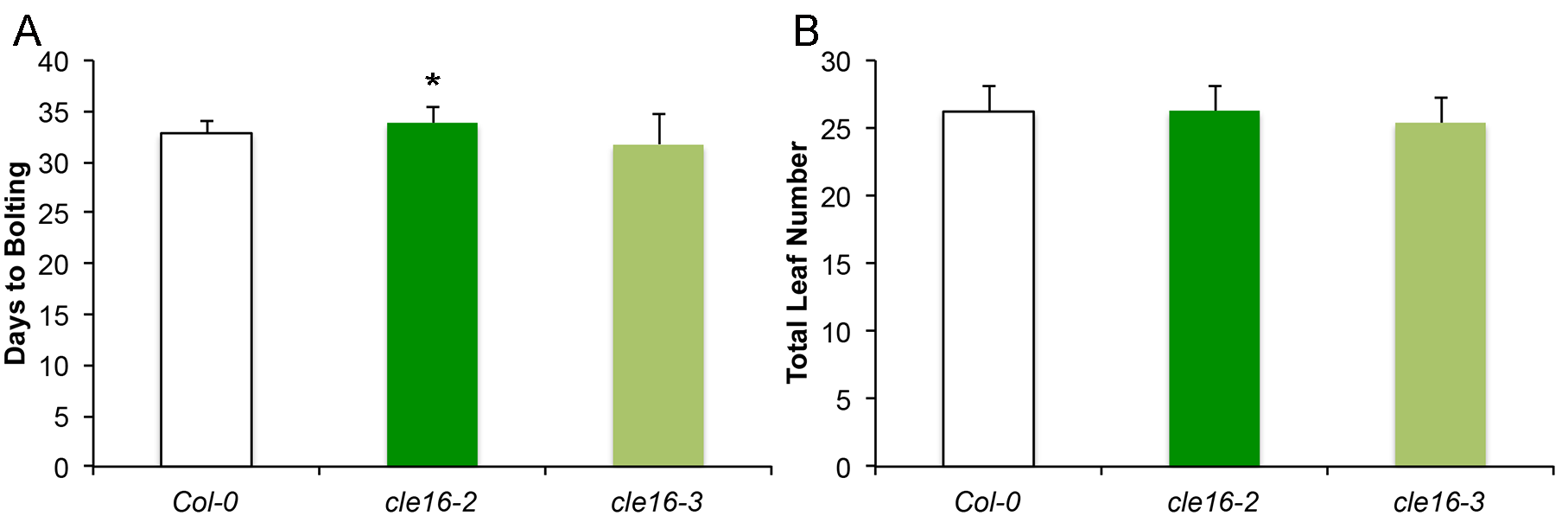

Supplement: S2 Fig — (TIF) [file pone.0202595.s002.tif]
