# Supplementary material for: The signaling peptide-encoding genes CLE16, CLE17 and CLE27 are dispensable for Arabidopsis shoot apical meristem activity
Source: PLoS One. 2018 Aug 16;13(8):e0202595. doi: 10.1371/journal.pone.0202595 (PMC6095548; doi:10.1371/journal.pone.0202595)
Supplement: S1 Table — (DOCX) [file pone.0202595.s003.docx]

**Table S1. Primer sequences used in the study.**

| **Name** | **Sequence** |
| --- | --- |
| Cloning |  |
| CLE16guideF | 5’-GATTGAACACAGCGGCGTAACCGG-3’ |
| CLE16guideR | 5’-AAACCCGGTTACGCCGCTGTGTTC-3’ |
| CLE17guideF | 5’-GATTGCTGCTCAATGGCCCACGAAA-3’ |
| CLE17guideR | 5’-AAACTTTCGTGGGCCATTGAGCAGC-3’ |
| Genotyping |  |
| CLE16CF | 5’-GAATCCAAAACCTGCTCTGC-3’ |
| CLE16CR | 5’-CGAAGGAGCAGTCAACACCT-3’ |
| CLE17CF | 5’-ACTCCTCCGGAACAAGGTTT-3’ |
| CLE17CR | 5’-CTTCTGCACGCACTTTCTCA-3’ |
| cle27LP | 5’-ATGACTCATGCTCGAGAATG-3’ |
| cle27RP | 5’-CTAGTTATGCAAAGGATCCG-3’ |
| cle27-2RP | 5’-TGACGAGTACCAAGAAGAAACG-3’ |
| SALK LBb1.3 | 5’-ATTTTGCCGATTTCGGAAC-3’ |
| sgRNA_FW | 5’-AGAAGAGAAGCAGGCCCATT-3’ |
| sgRNA_RV | 5’-TTCCCAAGGTCCAAAGACAC-3’ |
